# Supplementary material for: The aging factor EPS8 induces disease-related protein aggregation through RAC signaling hyperactivation
Source: Nat Aging. 2025 Sep 3;5(9):1750–70. doi: 10.1038/s43587-025-00943-w (PMC12443605; doi:10.1038/s43587-025-00943-w)
Supplement: Supplementary file 1 — Reporting Summary [file 43587_2025_943_MOESM1_ESM.pdf]

nature portfolio

Corresponding author(s): David Vilchez & Seda Koyuncu

Last updated by author(s): Jun 15, 2025

Reporting Summary

Nature Portfolio wishes to improve the reproducibility of the work that we publish. This form provides structure for consistency and transparency in reporting. For further information on Nature Portfolio policies, see our Editorial Policies and the Editorial Policy Checklist.

Statistics

For all statistical analyses, confirm that the following items are present in the figure legend, table legend, main text, or Methods section.

n/a	Confirmed	
☐	☒	The exact sample size (n) for each experimental group/condition, given as a discrete number and unit of measurement
☐	☒	A statement on whether measurements were taken from distinct samples or whether the same sample was measured repeatedly
☐	☒	The statistical test(s) used AND whether they are one- or two-sided<br>*Only common tests should be described solely by name; describe more complex techniques in the Methods section.*
☒	☐	A description of all covariates tested
☐	☒	A description of any assumptions or corrections, such as tests of normality and adjustment for multiple comparisons
☐	☒	A full description of the statistical parameters including central tendency (e.g. means) or other basic estimates (e.g. regression coefficient) AND variation (e.g. standard deviation) or associated estimates of uncertainty (e.g. confidence intervals)
☐	☒	For null hypothesis testing, the test statistic (e.g. F, t, r) with confidence intervals, effect sizes, degrees of freedom and P value noted<br>*Give P values as exact values whenever suitable.*
☒	☐	For Bayesian analysis, information on the choice of priors and Markov chain Monte Carlo settings
☒	☐	For hierarchical and complex designs, identification of the appropriate level for tests and full reporting of outcomes
☒	☐	Estimates of effect sizes (e.g. Cohen's d, Pearson's r), indicating how they were calculated

Our web collection on statistics for biologists contains articles on many of the points above.

Software and code

Policy information about availability of computer code

Data collection	No software was used for data collection.
Data analysis	Densitometry of filter trap and western blot assasys was quantified using ImageJ software (version 1.51). For motility assays, body bends were quantified using ImageJ software (version 1.53k) with the wrMTrck plugin (www.phage.dk/plugins).<br>We used GraphPad Prism (version 10.4.1) for statistical analysis of all the data. OASIS software (version 1) was used to determine mean lifespan (Yang, J.S., Nam, H.J., Seo, M., Han, S.K., Choi, Y., Nam, H.G., Lee, S.J. & Kim, S. OASIS: online application for the survival analysis of lifespan assays performed in aging research. PLoS One 6, e23525 (2011)).

For manuscripts utilizing custom algorithms or software that are central to the research but not yet described in published literature, software must be made available to editors and reviewers. We strongly encourage code deposition in a community repository (e.g. GitHub). See the Nature Portfolio guidelines for submitting code & software for further information.

Data

Policy information about availability of data

All manuscripts must include a data availability statement. This statement should provide the following information, where applicable:

- Accession codes, unique identifiers, or web links for publicly available datasets
- A description of any restrictions on data availability
- For clinical datasets or third party data, please ensure that the statement adheres to our policy

The authors declare that all data supporting the findings of this study are available within the paper and its supplementary Information files.

Research involving human participants, their data, or biological material

Policy information about studies with human participants or human data. See also policy information about sex, gender (identity/presentation), and sexual orientation and race, ethnicity and racism.

Reporting on sex and gender	N/A
Reporting on race, ethnicity, or other socially relevant groupings	N/A
Population characteristics	N/A
Recruitment	N/A
Ethics oversight	N/A

Note that full information on the approval of the study protocol must also be provided in the manuscript.

Field-specific reporting

Please select the one below that is the best fit for your research. If you are not sure, read the appropriate sections before making your selection.

☒ Life sciences ☐ Behavioural & social sciences ☐ Ecological, evolutionary & environmental sciences

For a reference copy of the document with all sections, see nature.com/documents/nr-reporting-summary-flat.pdf

Life sciences study design

All studies must disclose on these points even when the disclosure is negative.

Sample size

Exact sample sizes are provided in the corresponding Figure legends and Extended Data Figure legends.
No statistical methods were used to predetermine sample size; however, our sample sizes were selected based on standards established in the field and are similar to, or greater than, those reported to be sufficient in previous publications using the same procedures (i.e. lifespan, nose-touch, chemotaxis, motility, percentage of GABAergic neurodegeneration in C. elegans, percentage of activated caspase-3 in human neurons, filter trap assays, western blotting, and proteasome activity measurements):
- Koyuncu S et al, Nature 596:285-290 (2021)
- Llamas et al, Aging Cell 20: e13446
- Lee HL et al; Nature Metabolism 1: 790-810 (2019)
- Lee HL et al; Nature Aging 3:546-566 (2023)
- Koyuncu S et al, Nature Communications 9: 2886 (2018)
- Amrit FR et al, Methods 68: 465–475 (2014)
- Fatima A et al, Communications Biology 3: 262
- Alirzayeva H et al, Cell Reports 43: 114626 (2024)
- Segref A et al, Nature Communications 13: 5874 (2022))
- Koopman M et al, MicroPublication Biology: 10.17912/micropub.biology.000769 (2023)
- Fernandez-Abascal J, Neuron 110:470–485 (2022)
- Vilchez D et al, Nature 489:263-8 (2012)
- Vilchez D et al, Nature 489:304-8 (2012)
- Hart AC et al, Journal of Neuroscience 19, 1952-1958 (1999)
- Hahm JH et al, Nature Communications 6: 8919
- Liachko NF, Journal of Neuroscience 30, 16208-16219 (2010)

Data exclusions

For motility assays, worms were excluded from analysis if they showed less than 0.1 body-bends per second or were not recognized by the program. No data were excluded from other analyses.

Replication

At least three independent experiments were conducted for each assay to verify the reproducibility of the findings. If only two independent experiments were performed, this is indicated in the figure legend. All replication attempts yielded similar results. Lifespan assays were conducted at least twice, with 96 animals per condition. Exact sample sizes and the number of independent experiments are specified in the corresponding Figure and Extended Data Figure legends.

Randomization

For C. elegans experiments, worm populations were synchronized either by allowing young hermaphrodites to lay eggs for 6 hours or by using the bleaching method (PMID: 22710399). For synchronization via egg laying, young hermaphrodites were randomly picked from maintenance plates. For synchronization via bleaching, random chunks of agar containing mixed-stage animals were transferred from maintenance plates, and larvae were allowed to grow until a sufficient number of young hermaphrodites were available for bleaching. After the 6-hour egg-laying period or bleaching, the resulting larvae were raised to adulthood. For lifespan assays, adult worms were randomly picked and transferred from the synchronized population to the different experimental conditions. For all other experiments, adult worms were randomly distributed into the various experimental groups from single pulls of synchronized populations. The different experimental conditions were collected and lysed in a random order, although data collection and analysis were not randomized.

In the human cell line experiments, cells with similar confluence were split, and equal numbers of cells were transferred to new plates for the experiments. The plates were randomly assigned to different treatment conditions. Samples were collected and lysed in a random order, although data collection and analysis were not randomized.

Blinding

The samples and experimental conditions were not processed in a blinded manner by the researchers involved in this study. However, cells and worms were randomly allocated from from single pulls to different treatment conditions, and key experiments were independently repeated by different researchers to ensure reproducibility.
Filter trap, western blot, proteasome activity, and motility assays were not performed under blinded conditions, as these rely on objective instrument-based measurements and/or provide indirect quantitative outputs. Data analysis for these assays was likewise unblinded, as the investigators who performed the analysis also loaded the samples during the experiment, and the corresponding outputs from the measurement equipment were released in sequence.
For experiments with direct phenotypic outputs—such as lifespan assays, nose-touch assays, chemotaxis assays, quantification of GABAergic neurodegeneration in C. elegans, and activated caspase-3 levels in human neurons—blinding was not applied during data collection or analysis. This was due to the pronounced and well-characterized phenotypes associated with the disease models and treatment conditions used in this study. The researchers conducting these experiments had extensive prior experience with the specific strains and treatments, which often exhibit obvious phenotypes (e.g., severe defects in polyQ and ALS models, or smaller size in RAC RNAi-treated worms). Nonetheless, worms were randomly assigned to treatment groups, and experimental conditions were assessed in a randomized order. These assays and their analyses were also independently repeated by different researchers to confirm robustness and reproducibility.

Reporting for specific materials, systems and methods

We require information from authors about some types of materials, experimental systems and methods used in many studies. Here, indicate whether each material, system or method listed is relevant to your study. If you are not sure if a list item applies to your research, read the appropriate section before selecting a response.

Materials & experimental systems

n/a	Involved in the study	
☐	☒	Antibodies
☐	☒	Eukaryotic cell lines
☒	☐	Palaeontology and archaeology
☐	☒	Animals and other organisms
☒	☐	Clinical data
☒	☐	Dual use research of concern
☒	☐	Plants

Methods

n/a	Involved in the study	
☒	☐	ChIP-seq
☒	☐	Flow cytometry
☒	☐	MRI-based neuroimaging

Antibodies

Antibodies used

We used the following antibodies in this study:

*For western blot:
anti-α-tubulin (Sigma, T6199, 1:5,000. RRID: AB_477583). Monoclonal, clone number: DM1A
anti-β-actin (Abcam, ab8226, 1:1,000. RRID: AB_306371). Monoclonal, clone number: mAbcam 8226
anti-EPS8 (Proteintech, 12455-1-AP, 1:1000). Polyclonal
anti-FUS (Abcam, ab154141, 1:1000. RRID: AB_2885092). Monoclonal, clone number: CL0190.
anti-TDP43 (Abcam, ab225710, 1:1000). Polyclonal.
anti-USP4 (Abcam, ab181105, 1:1000). Monoclonal, clone number: EPR13846
anti-HTT (Cell Signaling, #5656, 1:1000). Monoclonal, clone number: D7F7
anti-EPS8L2 (Abcam, ab85960, 1:1,000, RRID: AB_1924963). Polyclonal.
anti-LGG-1 (PMID: 30910027, 1:2,000).
anti-LC3B (Cell Signaling, #2775, 1:1,000). Polyclonal.
anti-Phospho-RIP (Ser166) (Cell Signaling, #65746, 1:1,000). Monoclonal, clone number: D1L3S
anti-RIP (Cell Signaling, #3493, 1:1,000). Monoclonal, clone number: D94C12

*Filter trap of aggregation-prone proteins:
anti-GFP (AMSBIO, 210-PS-1GFP, 1:5,000. RRID: AB_10013682). Polyclonal.
anti-FUS (Abcam, ab154141, 1:1000. RRID: AB_2885092). Monoclonal, clone number: CL0190.
anti-TDP43 (Abcam, ab225710, 1:1000). Polyclonal.

*Immunocytochemistry:
anti-Cleaved Caspase 3 (Cell Signaling, #9661S, 1:300. RRID: AB_2341188). Polyclonal.
anti-MAP2 (2a+2b) (Sigma-Aldrich, #M1406, 1:300. RRID: AB_477171). Monoclonal, clone number: AP-20
Alexa Fluor 488 Goat anti-Mouse IgG (H+L) (ThermoFisher, A-11029, 1:500. RRID: AB_2534088). Polyclonal.
Alexa Fluor 568F(ab')2 Fragment of Goat Anti-Rabbit IgG (H+L) (ThermoFisher, A-21069, 1:500. RRID: AB_141416). Polyclonal.

* Protein immunoprecipitation:
anti-USP-4 antibody (Abcam, ab181105, 1:100)
anti-Normal Rabbit IgG (Cell Signaling, 2729, 1:378)

Validation

Validation of antibodies were done by the stated manufacturer's, this study, or previous publications and supported by the publications indicated in the manufacturer's website, the Resource Identification Portal (RRID) and other publications using C. elegans and human cells.
* anti-α-tubulin (Sigma, T6199, 1:5,000. RRID: AB_477583). The antibody was validated as a loading control for western blot analysis in C. elegans in our previous publications: PMID: 32451438; PMID: 27892468; PMID: 34172445; PMID: 34321666; PMID: 37118550
*anti-β-actin (Abcam, ab8226, 1:1,000. RRID: AB_306371) was used according to the manufacturer's instructions and our previous publications: PMID: 27892468; PMID: 30038412; PMID: 32451438; PMID: 37118550
* anti-EPS-8 (Proteintech, 12455-1-AP, 1:1000). The antibody was used according to the manufacturer's instructions, validated in previous studies (PMID: 34391775, PMID: 32147678), and confirmed by the data presented in this study (e.g. western blot of knockdown and overexpression experiments in human cells).
*anti-EPS8L2 (Abcam, ab85960, 1:1,000, RRID: AB_1924963). The antibody was used according to the manufacturer's instructions and validated in our previous publication (PMID: 34321666).
* anti-USP-4 (Abcam, ab181105, 1:1000). The antibody was used according to the manufacturer's instructions, validated in previous studies (PMID: 33038351, PMID: 29542252), and confirmed by the data presented in this study (e.g. western blot of knockdown experiments in human cells).
* anti-LGG-1 (PMID: 30910027, 1:2,000). This antibody was generated and validated in Springhorn, A. & Hoppe, T. Western blot analysis of the autophagosomal membrane protein LGG-1/LC3 in Caenorhabditis elegans. Methods Enzymol 619, 319-336 (2019).
* anti-LC3B (Cell Signaling, #2775, 1:1,000). The antibody was used according to the manufacturer's instructions, validated in previous studies (PMID: 39079530, PMID: 39635846).
* anti-Phospho-RIP (Ser166) (Cell Signaling, #65746, 1:1,000). The antibody was used according to the manufacturer's instructions, validated in previous studies (PMID: 39505876, PMID: 39526730).
* anti-RIP (Cell Signaling, #3493, 1:1,000). The antibody was used according to the manufacturer's instructions, validated in previous studies (PMID: 39681571, PMID: 39753884).
*anti-HTT (Cell Signaling, ab#5656, 1:1000). The antibody was used according to the manufacturer's instructions and validated in our previous publications (PMID: 30038412, PMID: 36611004).
* anti-GFP (AMSBIO, 210-PS-1GFP, 1:5,000. RRID: AB_10013682). This antibody has been validated for filter trap and western blot in C. elegans and human cells in our previous publications: PMID: 27892468; PMID: 30038412; PMID: 34172445; PMID: 34321666; PMID: 37118550.
* anti-FUS (Abcam, ab154141, 1:1000. RRID:AB_2885092) was used according to the manufacturer's instructions and our previous publications for filter trap and western blot experiments: PMID: 30038412; PMID: 34172445; PMID: 37118550.
* anti-TDP43 (Abcam, ab225710, 1:1000) was used according to the manufacturer's instructions and our previous publications for filter trap and western blot experiments where it was previously validated: PMID: 34172445; PMID: 37118550.
* anti-Cleaved Caspase 3 (Cell Signaling, #9661S, 1:300. RRID:AB_2341188) was used according to the manufacturer's instructions and validated in multiple studies and our previous publication (e.g. PMID:16736467, PMID:17099894, PMID:17299760, PMID:17990272, PMID:19830812, PMID:20235094, PMID:20593360, PMID:20653033, PMID:20653035, PMID: 37118550 etc.).
* anti-MAP2 (2a+2b) (Sigma-Aldrich, #M1406, 1:300. RRID:AB_477171) was used according to the manufacturer's instructions and validated in multiple studies and our previous publication (e.g. PMID:19058188, PMID:19950118, PMID:26509469, PMID: 37118550 etc)
*Alexa Fluor 488 Goat anti-Mouse IgG (H+L) (ThermoFisher, A-11029, 1:500. RRID:AB_2534088) was used according to the manufacturer's instructions and validated in multiple studies (e.g. PMID:34995520, PMID:35194846, PMID:35219381).
*Alexa Fluor 568F(ab')2 Fragment of Goat Anti-Rabbit IgG (H+L) (ThermoFisher, A-21069, 1:500. RRID:AB_141416) was used according to the manufacturer's instructions and validated in multiple studies (e.g. PMID:35111373, PMID:31526765, PMID:29103933).

Eukaryotic cell lines

Policy information about cell lines and Sex and Gender in Research

Cell line source(s)

In this study, we used the human HEK293 cell line (HEK293T/17) obtained from the American Type Culture Collection (ATCC). Catalog number: CRL-11268.
ALS-iPSCs (FUSP525L/P525L) were kindly provided by I. Bozzoni and A. Rosa (Sapienza University of Rome). This iPSC line was established and characterized for pluripotency in ref.: Lenzi J et al. ALS mutant FUS proteins are recruited into stress granules in induced pluripotent stem cell-derived motoneurons. Dis Model Mech 8: 755-766 (2015), (PMID: 26035390). ALS-iPSCs were raised from control iPSCs by TALEN (transcription activator-like effector nucleases)-directed mutagenesis and are homozygote for a FUS mutation (P525L) linked with severe ALS (Lenzi J et al. Dis Model Mech 8: 755-766 (2015)).

Authentication	The HEK293T/17 cell line commercially obtained from ATTC has not been authenticated in our laboratory. We have authenticated the iPSC lines in the laboratory by performing STR analysis (PMID: 30038412). We confirmed that the STR profile of the ALS-iPSCs used in this study matches with the profile of their isogenic control iPSCs.
Mycoplasma contamination	All the cell lines used in this study were tested for mycoplasma contamination at least once every 3 weeks. No mycoplasma contamination was detected.
Commonly misidentified lines (See ICLAC register)	None of the cell lines used in this paper are listed in the database of commonly misidentified cell lines maintained by ICLAC (version 12, released 16th January 2023)

Animals and other research organisms

Policy information about studies involving animals; ARRIVE guidelines recommended for reporting animal research, and Sex and Gender in Research

Laboratory animals

In this study, we used different Caenorhabditis elegans strains. For all the experiments, we used hermaphrodites worms.

Lifespan analysis was started from day 1 of adulthood. For all the other experiments on C. elegans, the specific age is indicated in the corresponding figures and/or figure legends.

The C. elegans strains used in this study were:

Wild-type (N2)
AM141 (rmIs133[unc-54p::Q40::yellow fluorescent protein (YFP)])
AM23 (rmIs298[F25B3.3p::Q19::CFP])
AM716 (rmIs284[F25B3.3p::Q67::YFP])
MAH602 (sqIs61[vha-6p::Q44::YFP + rol-6(su1006)])
CK405 (Psnb-1::TDP-43WT, myo-2p::dsRED)
CK423 (Psnb-1::TDP-43M337V, myo-2p::dsRED)
ZM5838 (hpIs223[rgef-1p::FUSWT::GFP])
ZM5842 (hpIs228[rgef-1p::FUSR522G::GFP])
ZM5844 (hpIs233[rgef-1p::FUSP525L::GFP])
DVG196 (rmIs284[F25B3.3p::Q67::YFP];sid-1(pk3321)V;uIs69[pCFJ90(myo-2p::mCherry) + unc-119p::sid-1])
VDL05 (eps-8(syb2901)IV))
VDL06 (eps-8(syb2901, syb3149)IV)
DVG365 (rmIs284[pF25B3.3::Q67::YFP]; eps-8(ok539))
DVG344 (rmIs284[pF25B3.3::Q67::YFP]); eps-8(syb2901)IV)
DVG345 (rmIs284[pF25B3.3::Q67::YFP]); eps-8(syb2901 syb3149)
DVG363 (rmIs133[unc-54p::Q40::YFP]); eps-8(syb2901)
DVG364 (rmIs133[unc-54p::Q40::YFP]); eps-8(syb2901, syb3149))
NFB2862 (Psnb-1::TDP-43WT, myo-2p::dsRED; juIs76[unc-25p::GFP + lin-15(+)]II)
NFB2863 (Psnb-1::TDP-43M337V, myo-2p::dsRED); juIs76[unc-25p::GFP + lin-15(+)]II)
NFB2858 (rmIs298[F25B3.3p::Q19::CFP]; otIs549[unc-25p::unc-25(partial)::mChopti::unc-54 3'UTR + pha-1(+)]; him-5(e1490)V)
NFB2859 rmIs284[F25B3.3p::Q67::YFP]; otIs549[unc-25p::unc-25(partial)::mChopti::unc-54 3'UTR + pha-1(+)]; him-5(e1490)V)
NFB2860 (hpIs223[rgef-1p::FUSWT::GFP); otIs549[unc-25p::unc-25(partial)::mChopti::unc-54 3'UTR + pha-1(+)]; him-5(e1490)V)
NFB2861 (hpIs233[rgef-1p::FUSP525L::GFP); otIs549[unc-25p::unc-25(partial)::mChopti::unc-54 3'UTR + pha-1(+)]; him-5(e1490)V)

Wild animals	The study did not involve wild animals.
Reporting on sex	In this study,we used hermaphrodites worms for all Caenorhabditis elegans experiments.
Field-collected samples	No field collected samples were used in the study.
Ethics oversight	We used the invertebrate C. elegans as a model organism and no ethical approval was required. According to the "Zentrale Kommission für die Biologische Sicherheit" (ZKBS), the responsible entity inside the Bundesamt für Verbraucherschutz und Lebensmittelsicherheit to assess the risk of Genetically Modified Organisms (GMO), genetic work with C. elegans is classified as risk group 1 (biological safety level 1: S1). Accordingly, we carried out our work on C. elegans in a S1-laboratory. The use of GMO in Germany is regulated by the "Gentechnik-Gesetz", and we followed the guidelines applying to S1 work with GMO (i.e., documentation of the project and of the, exact description of the creation and maintenance of the genetic modification or correct waste treatment).

Note that full information on the approval of the study protocol must also be provided in the manuscript.

Plants

Seed stocks	N/A
Novel plant genotypes	N/A
Authentication	N/A
