# Supplementary figures and images for: The aging factor EPS8 induces disease-related protein aggregation through RAC signaling hyperactivation
Source: Nat Aging. 2025 Sep 3;5(9):1750–70. doi: 10.1038/s43587-025-00943-w (PMC12443605; doi:10.1038/s43587-025-00943-w)

**Fig. 1a**

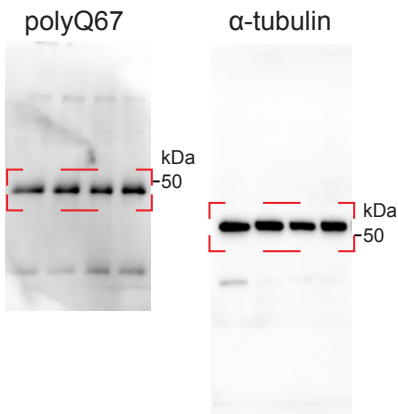

**Fig. 1d**

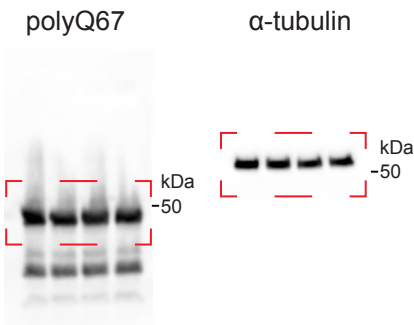

Supplement: Supplementary file 22 — Unprocessed western blots. [file 43587_2025_943_MOESM22_ESM.pdf]

**Fig. 2a**

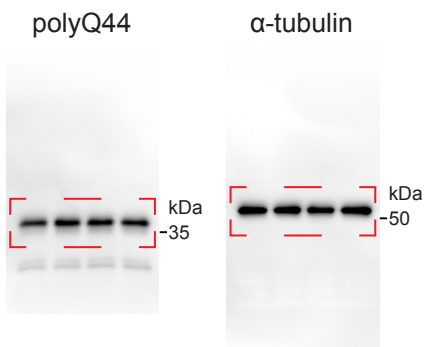

**Fig. 2b**

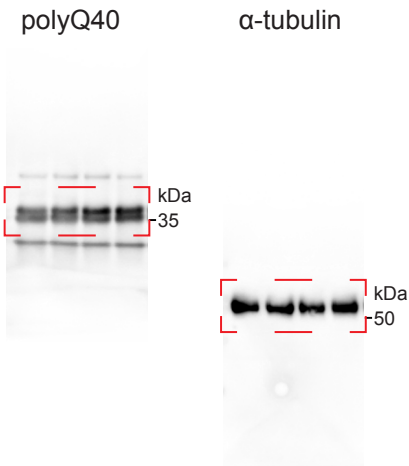

**Fig. 2d**

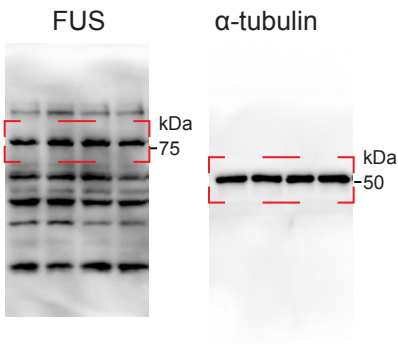

**Fig. 2e**

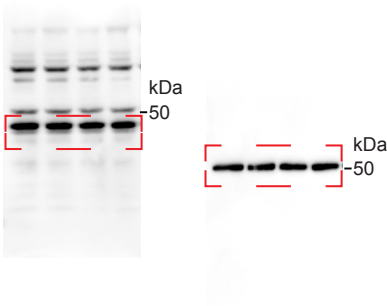

Supplement: Supplementary file 23 — Unprocessed western blots. [file 43587_2025_943_MOESM23_ESM.pdf]

**Fig. 3a**

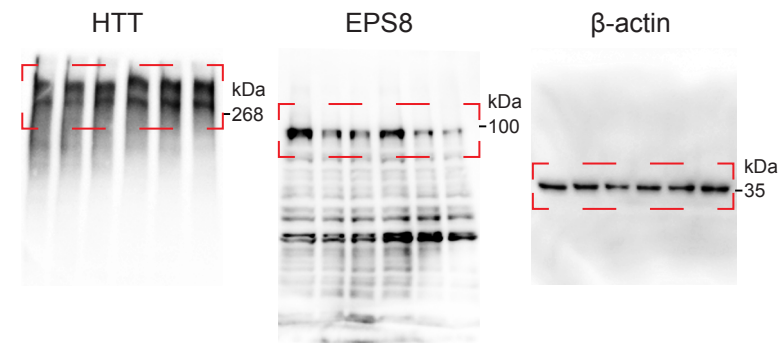

**Fig. 3b**

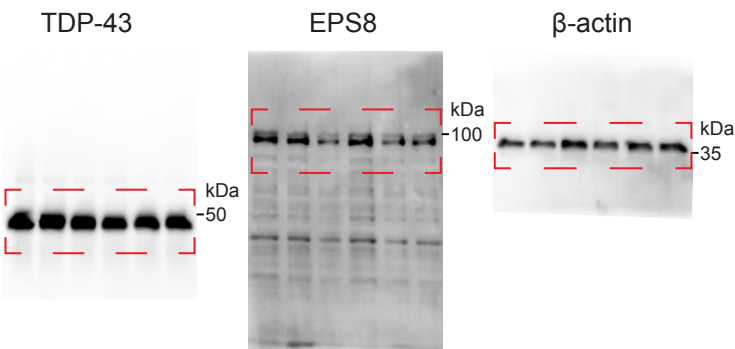

**Fig. 3c**

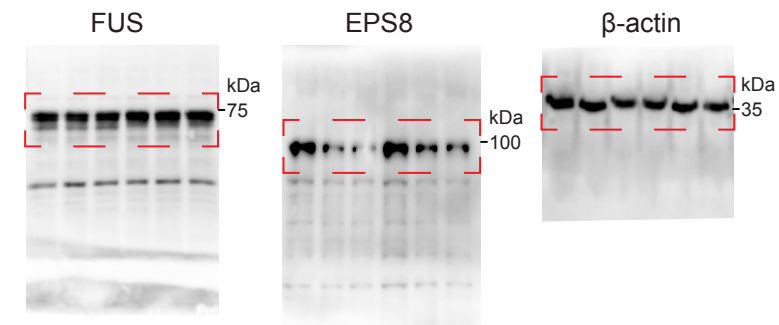

**Fig. 3e**

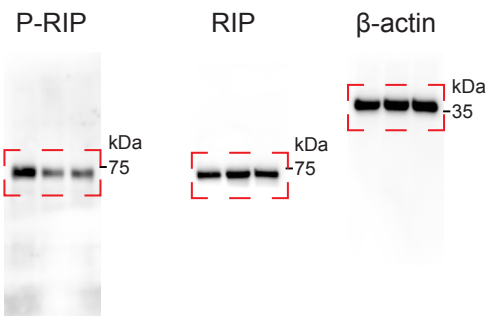

**Fig. 3f**

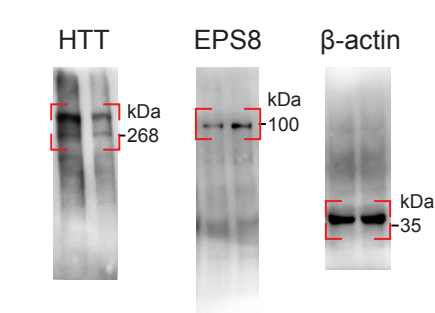

**Fig. 3g**

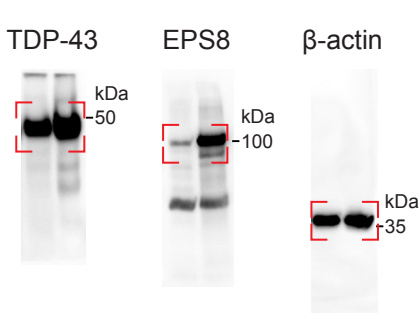

**Fig. 3h**

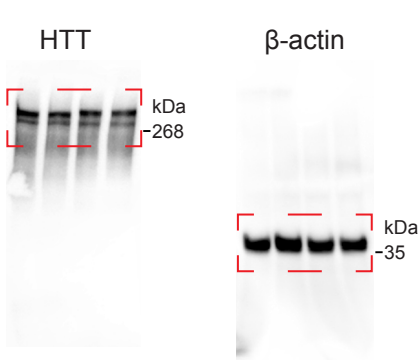

Supplement: Supplementary file 24 — Unprocessed western blots. [file 43587_2025_943_MOESM24_ESM.pdf]

**Fig. 4a**

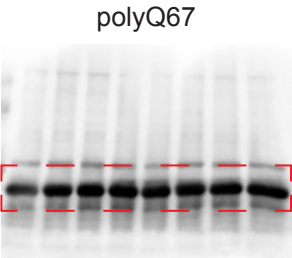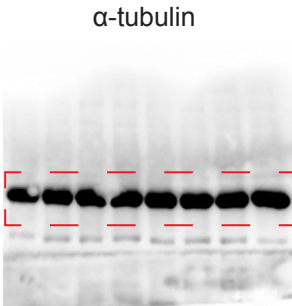

**Fig. 4b**

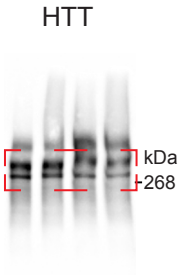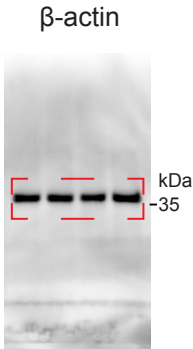

**Fig. 4c**

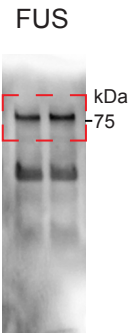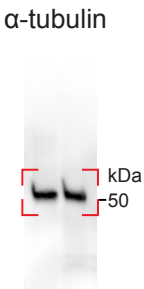

**Fig. 4d**

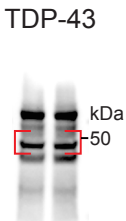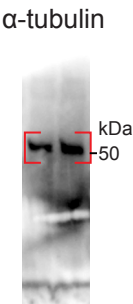

**Fig. 4e**

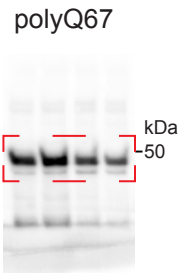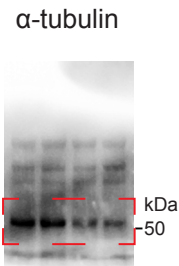

Supplement: Supplementary file 25 — Unprocessed western blots. [file 43587_2025_943_MOESM25_ESM.pdf]

**Fig. 5a**

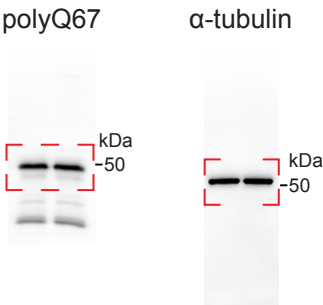

**Fig. 5b**

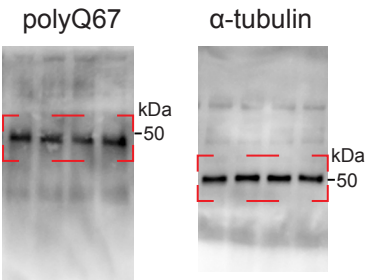

**Fig. 5c**

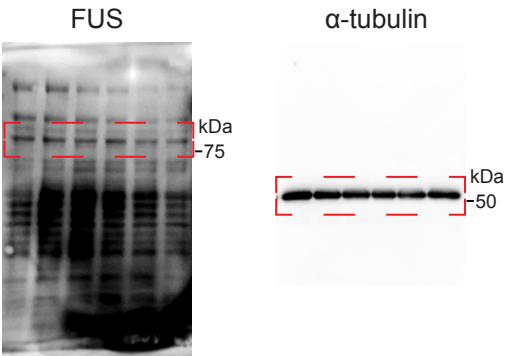

**Fig. 5d**

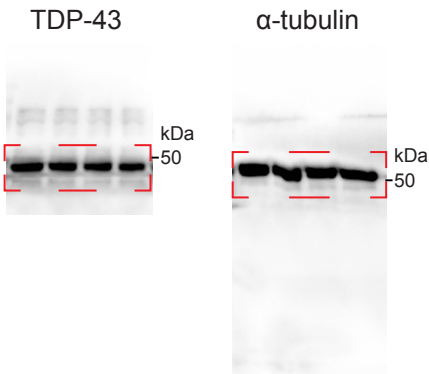

Supplement: Supplementary file 26 — Unprocessed western blots. [file 43587_2025_943_MOESM26_ESM.pdf]

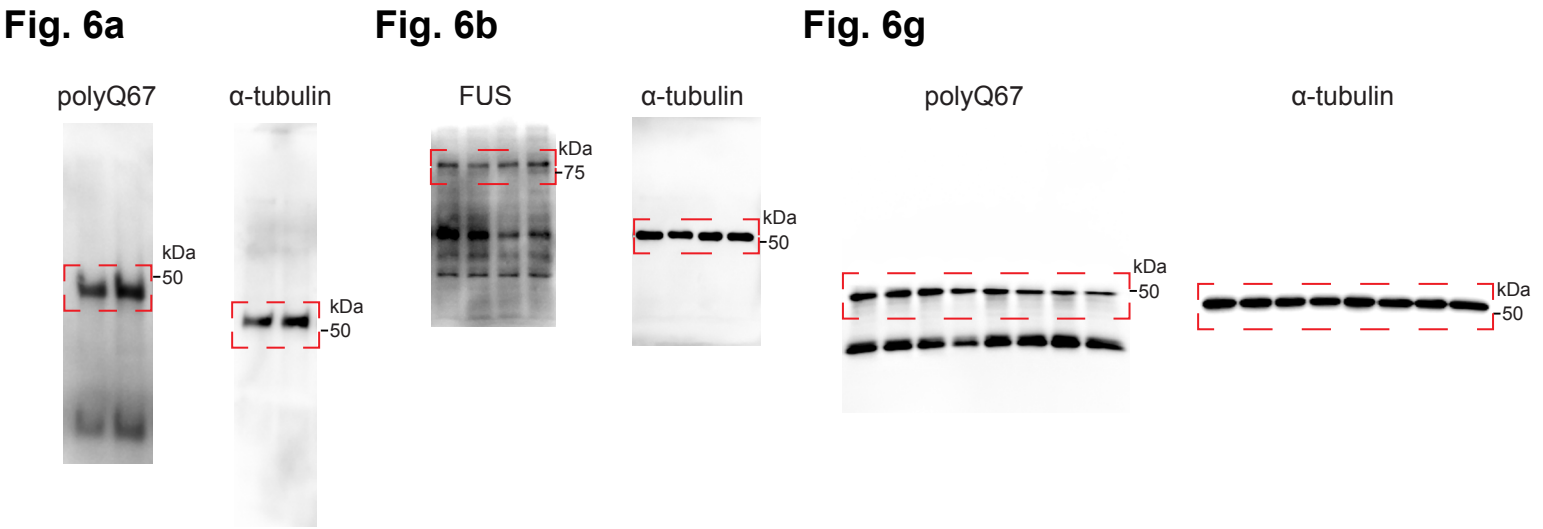

Supplement: Supplementary file 27 — Unprocessed western blots. [file 43587_2025_943_MOESM27_ESM.pdf]

**Fig. 7a**

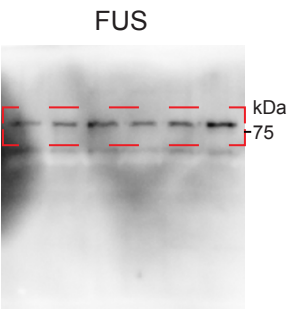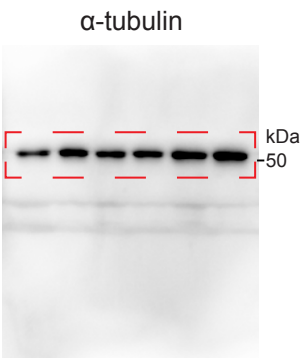

**Fig. 7b**

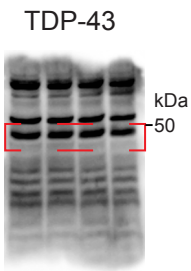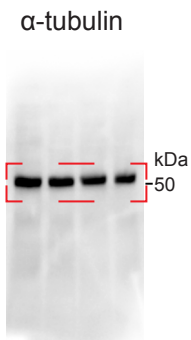

**Fig. 7h**

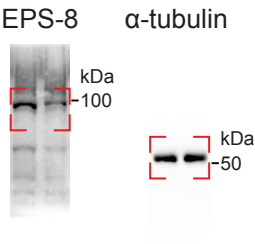

**Fig. 7j**

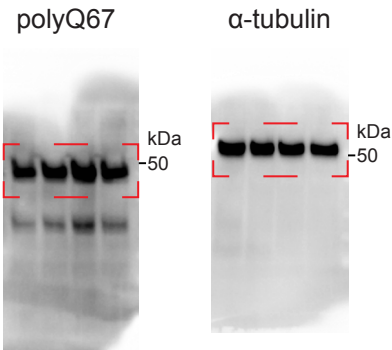

Supplement: Supplementary file 28 — Unprocessed western blots. [file 43587_2025_943_MOESM28_ESM.pdf]

**Fig. 8a**

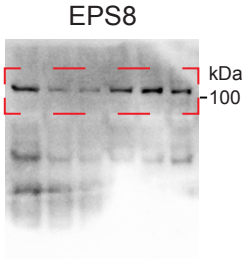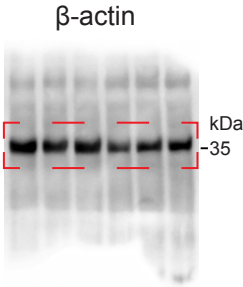

**Fig. 8b**

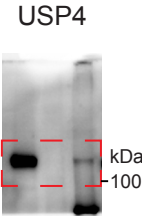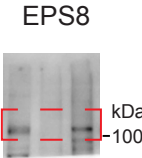

**Fig. 8c**

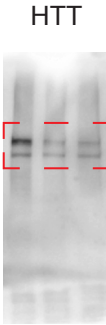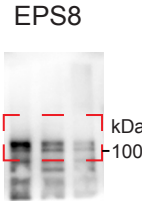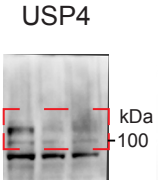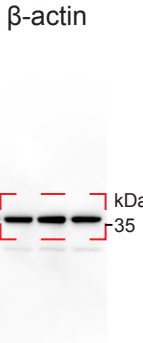

**Fig. 8d**

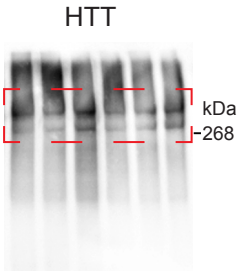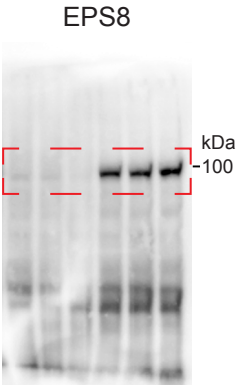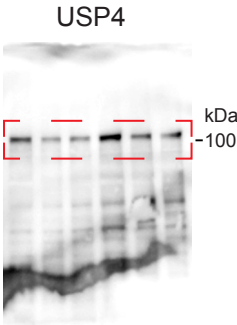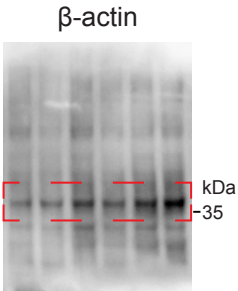

**Fig. 8e**

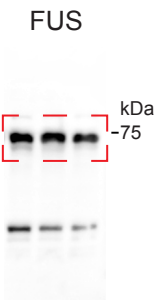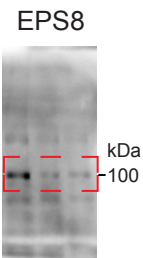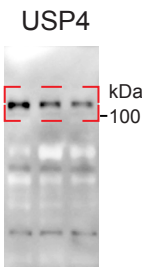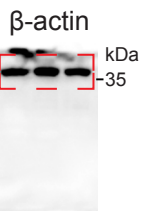

**Fig. 8f**

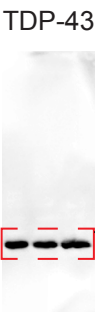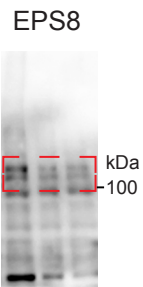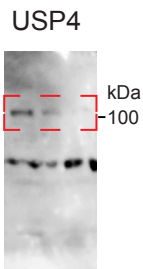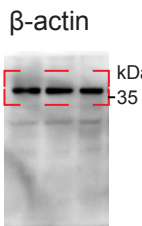

Supplement: Supplementary file 29 — Unprocessed western blots. [file 43587_2025_943_MOESM29_ESM.pdf]

**Extended Data Fig. 1a**

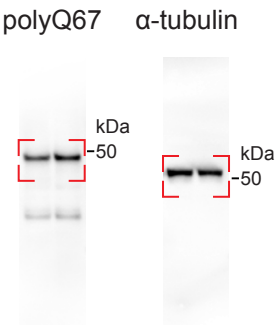

**Extended Data Fig. 1b**

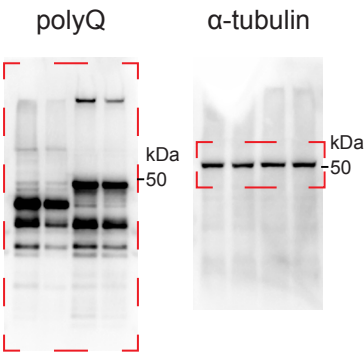

**Extended Data Fig. 1c**

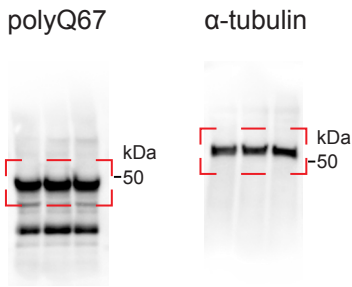

**Extended Data Fig. 1g**

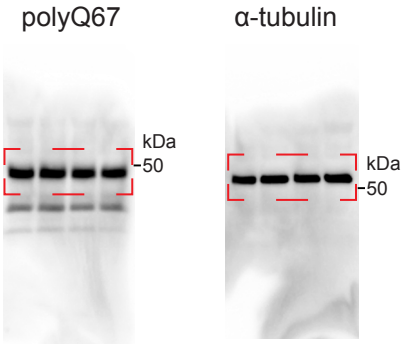

Supplement: Supplementary file 30 — Unprocessed western blots. [file 43587_2025_943_MOESM30_ESM.pdf]

Extended Data Fig. 3a

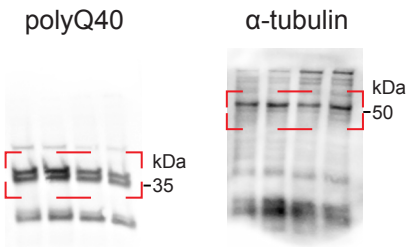

Supplement: Supplementary file 31 — Unprocessed western blots. [file 43587_2025_943_MOESM31_ESM.pdf]

Extended Data Fig. 5b

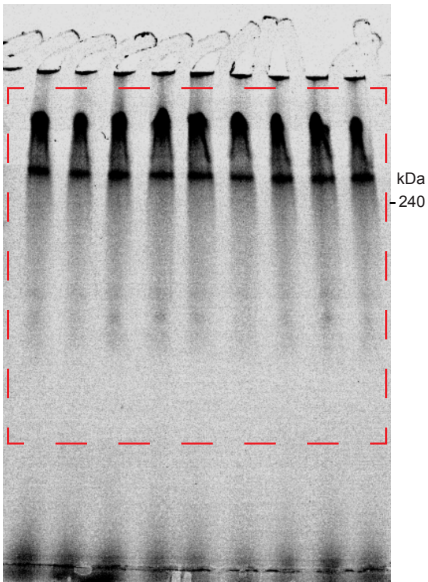

Supplement: Supplementary file 32 — Unprocessed gel. [file 43587_2025_943_MOESM32_ESM.pdf]

Extended Data Fig. 6b

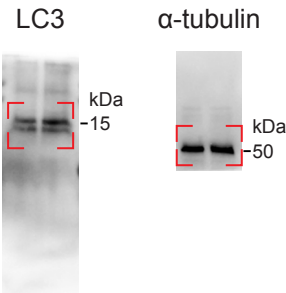

Extended Data Fig. 6e

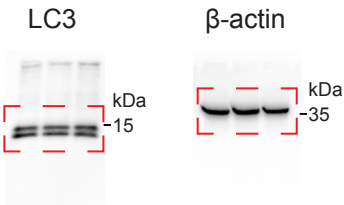

Extended Data Fig. 6f

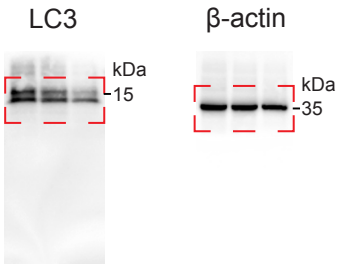

Supplement: Supplementary file 33 — Unprocessed western blots. [file 43587_2025_943_MOESM33_ESM.pdf]

Extended Data Fig. 7

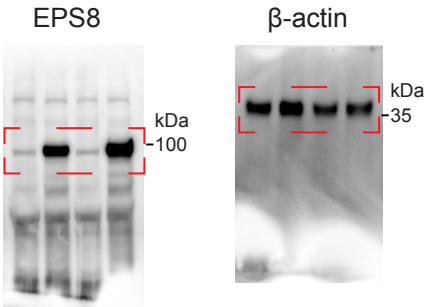

Supplement: Supplementary file 34 — Unprocessed western blots. [file 43587_2025_943_MOESM34_ESM.pdf]

**Extended Data Fig. 8a**

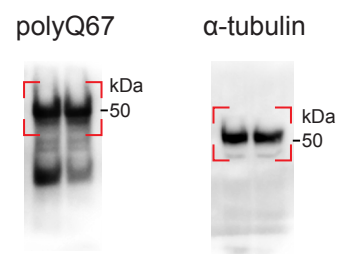

**Extended Data Fig. 8b**

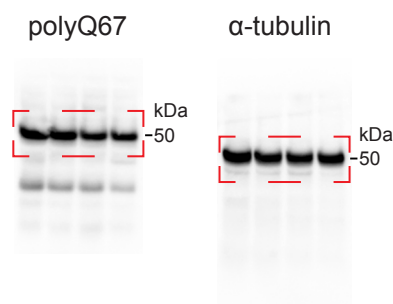

**Extended Data Fig. 8c**

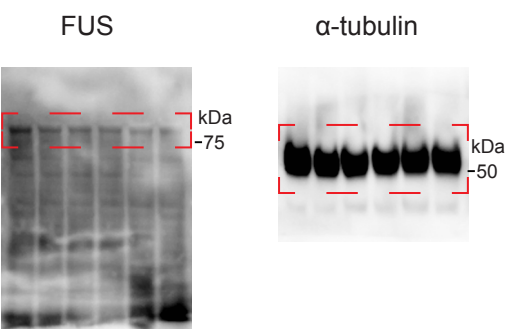

**Extended Data Fig. 8d**

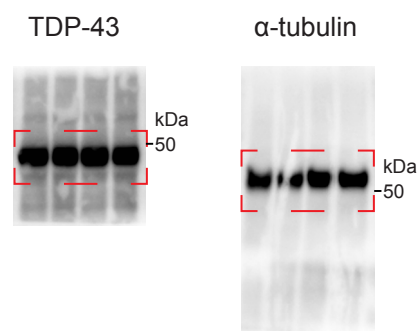

Supplement: Supplementary file 35 — Unprocessed western blots. [file 43587_2025_943_MOESM35_ESM.pdf]

Extended Data Fig. 10f

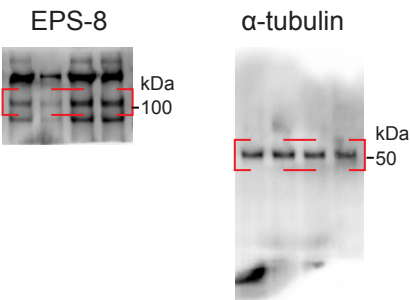

Supplement: Supplementary file 36 — Unprocessed western blots. [file 43587_2025_943_MOESM36_ESM.pdf]
